# A Study of the Relationship Between Students’ Global Perspective and Willingness to Communicate in English at an English Medium Instruction University in China

**DOI:** 10.3389/fpsyg.2022.873766

**Published:** 2022-07-22

**Authors:** Yuehai Xiao, Xiang Qiu

**Affiliations:** Department of English, Hunan Normal University, Changsha, China

**Keywords:** English as a global language, EMI universities, global perspective, willingness to communicate (WTC) in English, English as a foreign language (EFL) learning

## Abstract

Few previous studies have investigated the relationship between global perspective (GP) and willingness to communicate (WTC) in English. Hence, more studies are needed to validate their correlation. Furthermore, hardly any pertaining studies have been conducted at English Medium Instruction (EMI) universities. As such, the current study aimed to fill these gaps in the context of an EMI university in China, by investigating whether GP correlates with second language (L2) WTC and what factors impact the two variables. Data were collected from students *via* an online questionnaire (*n* = 315) and follow-up interviews (*n* = 11). The questionnaire findings confirmed a moderate positive correlation between GP and L2 WTC. The interview data unraveled that several factors influenced students’ L2 WTC, including needs and motivations driving L2 WTC, concerns constraining L2 WTC, and intercultural cognition facilitating L2 WTC. These findings suggest that: (A) students could be more determined to practice their English if they realize the significance of the role of English in their life; (B) teachers could foster students’ WTC by creating a non-threatening English-speaking environment and encouraging students to communicate in English in and outside the classroom; and (C) teachers could educate students about GP and L2 WTC, which might help to expand students’ horizon and stimulate their interests in foreign cultures and global affairs, so as to facilitate the sustainable growth of their English learning.

## Introduction

English is becoming widely used in numerous academic disciplines, and internationalization is coming into practice *via* “Englishization” of the curriculum among many higher education institutions (Galloway and McKinley, 2021). English as the medium of instruction (EMI) is defined as “the use of the English language to teach academic subjects (other than English itself) in countries or jurisdictions where English is not the first language (L1) of the majority of the population” (Macaro, 2018, p. 19). This definition echoes the current trends in the context of Chinese higher education, where there is a move from Chinese as the medium of instruction (CMI) toward English as the medium of instruction (EMI) at universities striving for internationalization (Rose et al., 2020).

EMI courses are expected to broaden students’ international horizons, to enable them to better understand and adapt themselves to the trend of globalization. However, not all students are proficient enough in English to keep up with academic instruction through it. And, in fact, not all subjects have been Englishized (Rose et al., 2020). While previous studies have investigated students’ global perspective (GP) or willingness to communicate (WTC) in English, the participants were either Chinese student sojourners or Chinese students who sometimes took English courses; little research has been done at EMI universities. Designed with optimal expectations, do EMI courses turn out to improve students’ GP? Given that these students spend more time in an English-speaking environment, are they more willing to communicate in English? The two questions need to be examined by more empirical studies. Hence, this study aims to investigate the relationship between students’ GP and WTC in English at an EMI university in China.

According to willingness to communicate in second language (L2 WTC) theory, if learners have a high WTC, they will take the initiative to practice using English as much as possible (MacIntyre et al., 1998; Braskamp et al., 2014; Alimorad and Farahmand, 2021). Therefore, English language teaching (ELT) should aim at fostering students’ L2 WTC.

Interpersonal and cross-cultural relationships have a profound impact on English as a foreign language (EFL) students’ growth. In a globalized and pluralistic world, people’s conceptualization of the relationship between themselves and others will often proceed from dependence through independence to interdependence (Braskamp et al., 2014). Braskamp et al. (2014) asserted that interpersonal development (the goal of which is interdependence), one of the three domains of GP, “is centered on one’s willingness to interact with persons with different social norms and cultural backgrounds, acceptance of others, and being comfortable when relating with others (p. 3).” For intercultural activities, it’s important for people to accept the cultural differences among those with different social backgrounds. With higher GP, the learners might have a higher level of tolerance and interest in people with various culture norms, which is echoed by Fang and Baker’s (2017) finding that GP can engage the learners in intercultural activities.

Fang et al. (2020) maintain that previous studies mostly focused solely on one aspect of foreign language WTC or global perspective, without sufficient empirical studies on whether there is a correlation between the two. Only a handful of previous studies (e.g., Yashima, 2002; Mahmoodi et al., 2013; Fang et al., 2020) have examined the correlation between the two variables GP and WTC, and the conclusion of the correlation between the two variables requires further empirical data to support it. There is a scarcity of studies on learners who are taught by foreign teachers through English and very few studies situated in EMI universities. In addition, the study of Fang et al. (2020) seemed to be the only one examining the differences among the four grades of undergraduate students, namely, freshmen, sophomores, juniors and seniors. Therefore, more diachronic research is needed to uncover the development of undergraduate learners’ GP and WTC.

In terms of theoretical significance, the current study will provide more empirical data on the correlation between GP and L2 WTC by expanding the sample size at an EMI university in China to confirm previous researchers’ findings. The pedagogical significance of this study is to generate innovative instruction based on the findings of this study, including the various factors impacting students’ WTC in English.

## Literature Review

### Theories of Willingness to Communicate and Global Perspective

#### Willingness to Communicate in Second Language

The model for L2 WTC in the process of foreign language teaching was proposed by MacIntyre et al. (1998), who believed that cultivating students’ willingness to communicate should be the goal of foreign language teaching. Hence, they formulated a six-level pyramid model, introducing various situational, and continuity factors that impact foreign language communication.

**FIGURE 1:**
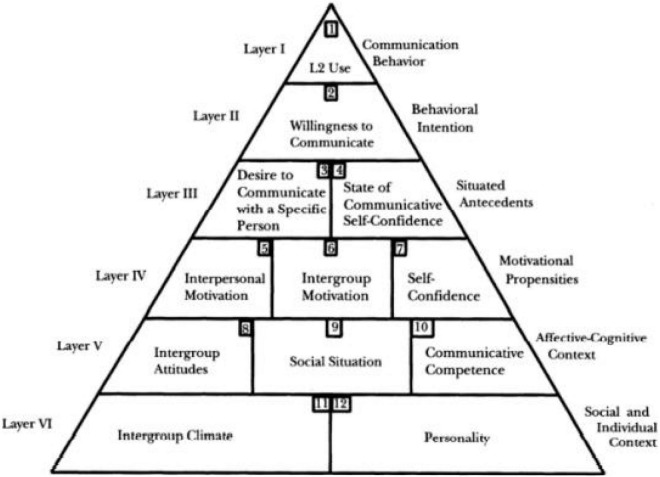
The pyramid L2 WTC model.

The pyramid model consisting of six layers is shown in Figure 1 (MacIntyre et al., 1998, cited in Fang et al., 2020).

#### Global Perspective

This study adopts the explanation of Braskamp et al. (2014), who described GP from a holistic perspective of global human development. Their explanation is based on two theoretical foundations. The first is the three domains of personal development proposed by Kegan (1994), namely, the cognitive, intrapersonal, and interpersonal aspects. King and Magolda (2005) later applied this model to the realm of intercultural maturity in terms of socio-cultural development. The second theoretical foundation encompasses cognitive, emotional and behavioral aspects of intercultural communication theory proposed by Chen and Starosta (1996). Braskamp et al. (2014) combined these two theories to discuss intercultural maturity and intercultural communication through cognitive, intrapersonal, and interpersonal concepts.

### Previous Studies

#### Previous Studies on Willingness to Communicate in Second Language

MacIntyre et al. (1998) regard WTC as a prerequisite to determine whether foreign language learners will eventually use their foreign language competence in various specific situations. At the same time, they admit that foreign language learners will be affected by many other lasting factors in the fourth to sixth layers of the WTC pyramid model. The results show that unless learners’ willingness to communicate can be improved at the same time, improving learners’ language skills and communicative competence is not enough to ensure learners’ ability to use foreign language in real conditions.

Studies about WTC, on the one hand, have focused on the WTC of different student populations. For instance, based on the theory of planned behavior, Zhong (2013) examined the L2 WTC of Chinese immigrants living in New Zealand and argued that, in the context of collaborative learning, learners’ willingness to communicate is only related to their behavioral beliefs. In combination with the theory of planned behavior, he also proposed a model of foreign language communication willingness and oral communication behavior. Lee and Zheng (2009) explored the characteristics of Chinese non-English major college students’ WTC in English and its relationship with learner factors through a questionnaire and correlation analysis. The results revealed that influenced by their English learning environment and Chinese cultural factors, these Chinese non-English majors had a low level of English communication willingness. Their cognitive styles tended to be passive, which led to their low awareness and ability to actively communicate in English.

Rashid et al. (2018) compared the WTC of two different groups of (English vs. non-English major) university students in China. A quite strong correlation between WTC in class and out of class was found in the English major group and moderate correlation in the non-English major group. Furthermore, it was revealed that these learners were lacking the opportunities of using English, particularly out of class. Factors affecting their WTC comprised their culture, interests and motivation.

In in-class, out-of-class, and digital contexts, Lee et al. (2022) investigated the L2 WTC of Korean and Taiwanese EFL students. They found that these Asian learners’ WTC can be enhanced by their exposure to English interaction opportunities, and the support from their teachers and their university. In blended learning context, Zhang et al. (2021) found Chinese EFL learners’ WTC to be dynamic and correlated with the interest, effectiveness, and difficulty of tasks.

On the other hand, some studies focused on the factors that have an impact on L2 WTC. Built on MacIntyre et al.’s (1998) theory, a number of researchers investigated some of the continuation factors in the pyramid model, which could mainly be classified into three kinds: intrapersonal (self-confidence, motivation, anxiety, etc.), environmental, and instructional (Chen, 2020).

In terms of instructional factors, it was found that teachers’ waiting time, error correction, selected topic, and help available to students can affect learners’ WTC (Zarrinabadi, 2014). L2 anxiety negatively correlated with L2 WTC in freshmen and sophomores. And students with higher levels of L2 anxiety had weaker WTC in English than those who were less anxious (Hu, 2016; Li, 2019).

An investigation on the WTC in English of three groups of Korean students unraveled that in the classroom, important predictors of L2 WTC included the foreign language learning motivation, confidence, and foreign language anxiety; outside the classroom, important predictors of L2 WTC included the students’ optional majors, levels of confidence in WTC in English, and foreign language anxiety; and in an online environment, student age, emotional variables, and network cross-cultural experience were found to be important predictors of language communication (Lee and Lee, 2019). A comprehensive analysis also unfolded a wide array of factors that affect the L2 WTC (Shirvan et al., 2019). Among them, language anxiety, motivation, and awareness of communication were most prominent.

However, the findings are not yet conclusive. For example, Mohammadian (2013) conducted a survey on the Iranian English learners and found shyness had no significant effect on students’ L2 WTC. Zhang et al. (2020) study underscored the individual differences rather than culture-related explanations of L2 WTC. They observed that individual differences in L2 WTC were dependent on openness to experience, and agreeableness, rather than extraversion.

The complex, dynamic interaction among individual, contextual, linguistic and educational factors may facilitate or impede L2 WTC of Iranian EFL tertiary students (Alimorad and Farahmand, 2021) and Chinese postgraduate students (Ma et al., 2022). More research is needed to uncover the difference between WTC in using English for general purposes and WTC in EMI settings (Ma et al., 2022).

#### Previous Studies on Global Perspective

Global perspective (GP) is a relatively new and comprehensive standard, which can be seen to cover international posture (Yashima, 2002) or similar concepts proposed by previous scholars. With the aim of accounting for age, ethnicity, race, and other identifying factors, Braskamp et al. (2014) designed a global perspective inventory (GPI) from 2007 to 2011. Study participants included undergraduate and graduate students from public and private schools. During this period, the researchers continuously modified the items of scales in the list according to the questionnaire results, put forward a total of seven versions of the list, and tested the reliability and validity of the questionnaire. In the end, an inventory on individual GP was obtained, in terms of cognitive, intrapersonal, and interpersonal aspects. Each of the three aspects included two scales, respectively, Knowing and Knowledge, Identity and Affect, Social Responsibility, and Social Interactions.

Studies on GP mainly fall into two categories, one of which is the investigation of GP from the perspective of learners’ intercultural citizenship. Byram (2008) characterized intercultural citizenship education as a combination of intercultural communicative competence and citizenship education. Nevertheless, in the field of ELT, particularly in EMI practice, there is a lack of the presence of Global Englishes and intercultural citizenship approaches (Baker and Fang, 2019).

Researchers have investigated the relationship between English language learning and use and the development of intercultural citizenship for Chinese international students. One study was conducted in a university located in southeast China (Fang and Baker, 2017) and the other in the context of United Kingdom higher education (Fang and Baker, 2021. The findings of the latter study demonstrated mostly positive attitudes toward intercultural citizenship, noting that “the awareness of being an international citizen could raise students’ motivation to experience foreign lives and study abroad.” In their study, the researchers argue that global citizenship is part of GP. These two studies could be regarded as previous studies focusing on GP to some extent.

Likewise, another study examined Chinese students’ perceptions about intercultural citizenship and its connection with language before, during and after studying in the EMI programs of an international university abroad (Baker and Fang, 2021). The participants seemed to develop intercultural citizenship with positive attitudes after their study, but some other study-abroad students had different perceptions. From the lens of EMI and intercultural citizenship, Baker and Fang (2021) proposed a pedagogy informed by Global Englishes and advocated such a critical pedagogy in ELT. Against the backdrop of globalization, participants considered English as a prerequisite to effectively engaging in intercultural communication. Nonetheless, there was a lack of data demonstrating the students’ awareness, motivation, and development of intercultural citizenship at international universities. Baker and Fang (2022) argued that English language teaching plays a pivotal role in developing the learners’ intercultural citizenship and called for more research in this area.

One study along this line explored Thai and Chinese students’ perceptions on their development of intercultural citizenship in EMI programs and examined the relationship between intercultural citizenship and English in international universities (Boonsuk and Fang, 2021). The participants generally displayed positive attitudes toward intercultural citizenship and they believed that living in a foreign country may promote intercultural awareness. Furthermore, their intercultural citizenship competencies could be enhanced by English proficiency and regular interaction with international friends.

On the other hand, another category of studies on GP focused on narrowing it down to specific abilities, scales and so on. Li (2011) defined her own “global vision” as a broad, transnational, and comprehensive position from which students can macroscopically view international and transnational texts. She adapted this concept from the National English Curriculum (NEC) in China and conducted a hypothesis experiment in which students were introduced to foreign cultures through foreign movies, speeches, experts, and English corners in order to demonstrate whether their interest in foreign cultures and awareness of cultural differences improved. This study could be considered as somewhat abstract and subjective. Because unlike Braskamp et al. (2014) study, this investigation lacked specific criteria from several detailed scales to define the concept of GP. And students’ improvement was not evaluated by scores.

Liu et al. (2015) explored the extent to which each aspect of global perspective was influenced by three types of online open classes: humanities, natural sciences, and social sciences. They defined GP as people’s ability to understand the history of the world and the current international community from a global perspective, evaluate the status and role of their own countries, recognize their rights and obligations, and demonstrate appropriate behaviors and attitudes in international communication. GP in their study fell into three scales: awareness, knowledge, and ability, each of which involved several specific activities or attitudes.

Zhang and Zhu (2020) analyzed the results of the 2018 Program for International Student Assessment (PISA) global competency assessment and made four suggestions about developing Chinese students’ global competency. PISA defines global competence as the ability to “analyze local, global and cross-cultural issues, understand and appreciate the perspectives and world views of others, interact openly, appropriately and effectively with people from different cultural backgrounds, and be able to take action for collective wellbeing and sustainable development.” Based on this definition, PISA divides global competency into four interconnected dimensions. Even though their study did not include a clear definition of GP, the connotations of global competency could be viewed as the components of GP.

While the following two studies did not directly address the relationship between GP and L2 WTC, they discussed the relationship among Global Englishes, EMI, and English pedagogy. To some extent, they can be considered as investigating the connection between GP and L2 WTC, because “a Global English-informed pedagogy “focuses on the authentic use of English in international situations to (re)construct and (re)negotiate meaning for intercultural communication” (Fang and Ren, 2018, p. 385). To use English in authentic international situations requires intercultural knowledge, which is part of GP.

Liu and Fang (2022) examined the NEC in China regarding phonology and cultural learning informed by Global Englishes. They argued that there is enormous difference between language taught in a classroom and language used in daily life. And “Global Englishes” are yet to be introduced to English language classes properly. In the contexts of two universities in Macau and the Chinese mainland, Cai and Fang (2022) found that while some teachers used different translanguaging strategies, other teachers were reluctant to practice translanguaging.

#### Previous Studies on the Relationship Between Willingness to Communicate in Second Language and Global Perspective

In the literature, three articles explored the relationship between global perspective and WTC in English. Yashima (2002) proposed the concept of “international posture” when examining the factors that affect Japanese students’ WTC in English. The findings revealed that international posture affects the motivation at the fourth level in the pyramid model; thus, it can be used to predict second language communication proficiency and confidence.

Drawing from “intergroups attitude” in the fifth layer of the pyramid model, Yashima’s (2002) “international posture” describes the extent to which individuals are “interested in foreign or international affairs, willing to live or work abroad, willing to work with cross-cultural interaction partners, hope to open to different cultures or the ethnocentric attitude.” It can be further divided into the following four components: interest in international vocation/activities (IVA), intercultural friendship orientation (IFO), interest in foreign affairs (IFA), and intergroup approach avoidance tendency (AAT).

The current study maintains that the four aspects of IVA, IFO, IFA, and AAT proposed by Yashima (2002) belong to the cognitive domain, intrapersonal domain and interpersonal domain of GP. Overall, “international posture” can be regarded as part of the definition and components of GP adopted in this study; the “international posture” demonstrated in Yashima’s study directly affects L2 WTC, which indirectly indicates that GP affects L2 WTC.

Mahmoodi et al. (2013) discovered that WTC and international posture were positively correlated. Fang et al. (2020) also found a weak positive correlation between GP and WTC in English. Nonetheless, they called for more research on this correlation. Another gap in the literature is that, little is known about the differences among the GP and L2 WTC of the four grades of undergraduates at EMI universities in China. To what extent do the four groups differ? The third gap is that the diverse factors impacting GP and L2 WTC require further exploration. Furthermore, previous findings about some factors remained inconclusive. For instance, L2 anxiety was found to cause weaker WTC in English (Lee and Lee, 2019; Shirvan et al., 2019). However, some other researchers argued that L2 anxiety did not significantly affect students’ L2 WTC (e.g., Mohammadian, 2013; Zhang et al., 2020).

### Research Questions

To address the gaps in the literature, this study seeks to answer the following research questions:

Q1. What are the differences among the GP and L2 WTC of freshmen, sophomores, juniors, and seniors of an EMI university in China?
Q2. Is there a correlation between the GP and L2 WTC of students at the EMI university?
Q3. What are the factors that might influence students’ global perspective and WTC in English?

## Materials and Methods

### Research Context

This study was conducted at an EMI university in southern China in Spring 2021. The university was founded approximately 15 years ago thanks to a collaboration between a top-30 university in Mainland China and a university in Hong Kong. Its vision is to foster the whole-person development of the students and equip them with international perspectives.

The university has been recruiting professors and lecturers from prestigious universities all over the world. And most courses (except for Chinese culture) are taught in English. Intercultural interactions are encouraged through exchange programs with universities abroad. It has established partnerships with many foreign universities, providing students with rich opportunities for experiencing foreign culture and education. About 65% of its undergraduates choose to pursue further study abroad, which might affect these students’ WTC in English and GP as a result of the university’s educational philosophy and management.

### Participants

The participants in this study were non-English majors from year one to year four at an EMI university. For the questionnaire, the respondents involved 315 students from four grades: freshmen (44 students, 13.97%), sophomores (137 students, 43.49%), juniors (83 students, 26.35%), and seniors (51 students, 16.19%). Students’ majors comprised Science, Business, Engineering, Liberal Arts, English, and Medicine. Most of the courses they took were EMI courses, except for several Chinese-related courses.

For the interview, the number of the students who volunteered to provide their contact information was more than the current study needed, and potential interviewees were selected mainly based on the time they spent and their mean scores on the GP and L2 WTC scales. Eleven students, including four freshmen, two sophomores, two juniors, and three seniors participated in the follow up interviews. Assuming that students with higher English proficiency may have higher L2 WTC, this study chose participants whose English proficiency was in the middle 33% or upper 33% of their class. Four of the 11 interviewees were male and seven were female.

### Instruments

#### Questionnaire

In this study, we adopted and revised Fang et al.’s (2020) two questionnaires, which were based on the Global Perspective Inventory (Research Institute for Studies in Education, 2017) and L2 WTC questionnaire (Mystkowska-Wiertelak and Pawlak, 2017) respectively. The two questionnaires of this study consisted of 20 items in total, with 12 concerning the GP scale and 8 concerning the L2 WTC scale. Each item was rated on a 6-point Likert scale. Since participants with different English proficiency may have different understandings of the questionnaires in English, they were translated into Chinese.

The questionnaires were administered online using a tool called Sojump (*Wenjuanxing* in Chinese). In this way, answers from large numbers of students at different universities and schools could be collected conveniently. In the original version conducted on paper in real classrooms, numbers were displayed for participants to choose from, and each number corresponded to an opinion. In this online version, to mitigate the possibility of participants relating the numbers to their scores, numbers were not shown beside opinion options. That way, scores were assigned to each choice implicitly.

For items on the GP scale, the original choices were “strongly disagree,” “disagree,” “neither agree nor disagree,” “agree,” and “strongly agree.” Chinese students may have the tendency to choose the middle choice. In order to avoid that, the original five choices were changed to the following six progressive options: “Never true of me,” “Usually true of me,” “Somewhat not true of me,” “Somewhat true of me,” “Usually true of me,” and “Always true of me.”

#### Interviews

The interview questions were also adapted from those used by Fang et al. (2020). Because participants from the EMI university were located in a different city than the interviewers, to reduce the difficulty of data collection, all interviews were conducted on WeChat. Table 1 shows the background information of the interviewees.

**TABLE 1:** Participants’ background information.

Participant ID	Grades	Gender	Majors	Level of English proficiency in class
A		Female	Business	
B		Male	Business	
C	Freshmen	Female	Science	Middle 33%
D		Female	Business	
	
E	Sophomores	Male	Science	
F		Female	Engineering	
	
G	Juniors	Female	Science	
H		Female	Liberal Arts	
	
I		Male	Business	
	
J	Seniors	Female	Business	Upper 33%
K		Male	Business	

Interviews were conducted following questionnaire analysis, the results of which demonstrated that seniors had the highest mean scores on both the GP and L2 WTC scales. To discover the factors that contributed to this finding, a question (*Do you think your willingness to communicate with others in English has increased or decreased in your senior year? Why is that?*) was added for the three senior interviewees only. Apart from that, all interviewees were asked the same questions and answered them mostly by voice messages. Follow-up questions were asked to probe into participants’ answers and previous experience. In doing so, this study collected the facts and opinions concerning the interviewees’ GP and WTC in English, with the aim of uncovering the relationship between the two variables.

## Findings

### Questionnaire Findings

The current study used SPSS Version 20.0 to analyze the questionnaire data through the Cronbach’s α coefficient reliability, the means of participants’ GP and L2 WTC scales across the four groups, a one-sample Kolmogorov-Smirnov test (to determine normal distribution of data), a one-way Analysis of Variance (ANOVA), as well as a Pearson Correlation Analysis.

To begin with, the GP and L2 WTC scales’ reliability, the Cronbach α was 0.743 and 0.893, respectively, both of which were higher than 0.70. Therefore, the two questionnaires were considered to have high internal consistency.

The first research question is about the differences of GP and L2 WTC among the students of the four groups. Table 2 shows the means of the scores of the two questionnaires’ four groups ranging from freshmen to seniors. As for the mean value of GP questionnaire, the lowest value is 4.21 of the junior group, and the highest value is 4.25 of the senior group. As for the mean value of L2 WTC questionnaire, the lowest value is 4.20 for freshmen and the highest value is 4.48 for seniors.

**TABLE 2:** Means of GP and L2 WTC of students of the four groups.

		*n*	*M*
GP	Freshmen	44	4.2311
	Sophomores	137	4.2251
	Juniors	83	4.2199
	Seniors	51	4.2533
	Total	315	4.2291
L2WTC	Freshmen	44	4.2045
	Sophomores	137	4.4571
	Juniors	83	4.3946
	Seniors	51	4.4804
	Total	315	4.4091

The highest mean values of the two questionnaires both fell into seniors. Why was that? This is the question to be further explored in the follow-up interviews.

In Table 3, the asymptotic significance (2-tailed) of the mean scores of GP questions and L2 WTC questions in the questionnaire were 0.221 and 0.388, both of which are higher than 0.05, indicating that the data had normal distribution. Therefore, it was suitable to run a one-way ANOVA.

**TABLE 3:** Results of the one-sample Kolmogorov-Smirnov test.

		GP *M* for all participants	L2 WTC *M* for all participants
N		315	315
Normal parameters^a,b^	*M*	4.2291	4.4091
	*SD*	0.54465	0.86218
Most extreme differences	Absolute	0.059	0.051
	Positive	0.050	0.047
	Negative	−0.059	−0.051
Kolmogorov-Smirnov Z		1.049	0.903
Asymp. Sig. (2-tailed)		0.221	0.388

*^a^Test distribution is normal.*

*^b^Calculated from data.*

As Table 4 shows, the results of one-way ANOVA indicated that there was no significant difference (*p* = 0.988 > 0.05) between the means of the GP scale of the four groups of participants. Neither was there significant difference (*p* = 0.353 > 0.05) of the means of the L2 WTC scale across the four groups. Namely, participants from each group demonstrated similar GP and L2 WTC.

**TABLE 4:** Results of one-way ANOVA across the four groups.

		*df*	*F*	*p*
GP Mean	Between groups	3	0.044	0.988
	Within groups	311		
	Total	314		
L2WTC Mean	Between groups	3	1.092	0.353
	Within groups	311		
	Total	314		

**FIGURE 2:**
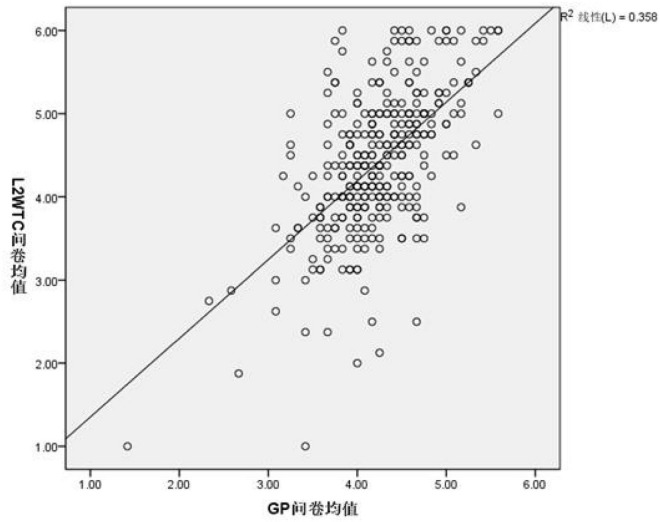
Scatter plot between the means of GP and L2 WTC.

The second research question intended to determine the correlation between GP and L2 WTC. A moderate positive correlation was identified in Figure 2, as the scatter plot drew close to the line and was symmetrically distributed around it, with an upward trend, which is more distinct than the trend in Fang et al.’s (2020) scatter plot.

Table 5 shows that the significance = 0.000 < 0.01; thus, GP and L2 WTC were significantly correlated. The correlation value (= 0.598) between GP and L2 WTC scales was in the range of 0.5 − 0.8, which indicates moderate correlation according to the statistic criterion (0.3 < *r* < 0.5, low correlation; 0.5 < *r* < 0.8, medium correlation; 0.8 < *r* < 1, high correlation). Therefore, the two variables GP and L2 WTC were considered to have medium correlation.

**TABLE 5:** Results of the pearson correlation analysis of the means of GP and L2 WTC.

		GP mean	L2WTC mean
GP Mean	*p*	1	0.598**
	Asymp. Sig. (2-tailed)		0.000
L2WTC Mean	*p*	0.598**	1
	Asymp. Sig. (2-tailed)	0.000	

**Correlation is significant at the 0.01 level (2-tailed).

### Interview Findings

Table 6 shows the sum and mean scores of the interviewees’ and all the survey participants’ GP and L2 WTC. As for the GP overall score, all interviewees except for A, E, H, and J, scored higher than 48, meaning they chose at least “somewhat true of me” for the 12 items. As for L2 WTC overall score, all interviewees except for A and B scored more than 32, meaning that they chose at least “perhaps willing” for the 8 items.

**TABLE 6:** Interviewees’ scores of GP and L2 WTC.

Participant	Grade	GP			L2 WTC		
		Overall score (max. 72)	Individual *M* scores	*M* per grade	Overall score (max. 48)	Individual *M* scores	*M* per grade
A	Fresh.	45	3.7500	4.2311	26	3.2500	4.2045
B		52	4.3333		31	3.8750	
C		64	5.3333		37	4.6250	
D		57	4.7500		40	5.0000	
E	Soph.	47	3.9167	4.2251	35	4.3750	4.4571
F		48	4.0000		36	4.5000	
G	Juniors	49	4.0833	4.2199	44	5.5000	4.3946
H		44	3.6667		44	5.5000	
I	Seniors	50	4.1667	4.2533	35	4.3750	4.4804
J		46	3.8333		40	5.0000	
K		59	4.9167		47	5.8750	

In general, seniors scored more highly on both GP and L2 WTC. For instance, participant A (a freshman) had the lowest mean score among the 11 interviewees, while participant K (a senior) had the highest sum score of GP and L2 WTC. Individually, however, some students from lower years had higher scores than students from higher grade levels. For example, as a freshmen, participant C had the highest scores on GP; whereas a senior, participant I, had the second-lowest mean score on L2 WTC. However, their scores did not necessarily reflect their overall GP or L2 WTC. Data from the interviews supplement these findings to provide a holistic view of participants’ GP and L2 WTC.

The interview data were coded over multiple rounds. Based on the codes assigned to the interview data, sixteen themes (as shown in Table 7) were identified. These themes were further categorized into three types: (1) Needs and motivations driving L2 WTC, (2) Concerns constraining L2 WTC, and (3) Intercultural cognition facilitating L2 WTC.

**TABLE 7:** Factors influencing L2 WTC.

Needs and motivations driving L2 WTC	Concerns constraining L2 WTC	Intercultural cognition facilitating L2 WTC
Theme 1: In-class interactions and discussion with teachers or peers Theme 2: Needs for final year project or master’s program applications Theme 3: IELTS or GMAT Theme 4: Need to improve interpersonal relationships Theme 5: Willingness to improve oral English Theme 6: Current or potential job requirements	Theme 7: Lack of confidence in accent, grammar, vocabulary Theme 8: Peer pressure in out-of-class social contexts (e.g., being seen as showing-off and awkwardness speaking English among Chinese-speakers) Theme 9: Awareness of avoiding taboos Theme 10: Awareness of the western-centrism and promotion of their values	Theme 11: Possible needs in present life or when living abroad Theme 12: Interests in the culture of English-speaking countries, such as movies, music, slangs, brain teasers Theme 13: Willingness to help foreigners Theme 14: Awareness of the role of English as a lingua franca Theme 15: Accessing firsthand sources of information, such as news or comments on foreign websites Theme 16: Understanding GP

#### Needs and Motivations Driving Willingness to Communicate in Second Language

Theme 1: In-class interactions and discussion with teachers or peers

All interviewees, more or less, mentioned the need to communicate with teachers and peers in their EMI classes. Since their admission to the EMI university, most of the courses these students had taken were taught through English by teachers from foreign countries or Hong Kong, who speak limited Chinese. If they want to catch up with the teacher, understand what the teacher said, and ask or answer questions, they have no choice but to use English. We discovered this by asking interviewees “How often do you use English to communicate with others? Under what circumstances? Would you mind giving some examples?” When facing this question, participant E said he typically uses English twice a week to talk with his English teachers in or after classes, and he predicted he would use English frequently in his higher level learning.

Participant H commented that she used English “about four times per week, because our school is an EMI school, and there are many foreign teachers, so I basically have to communicate with foreign teachers every day.” In her eyes, the process of taking EMI courses was similar to the process of learning English, through which she picked up new words and grammar.

Theme 2: Needs for final year project or master’s program application

In addition to interacting with teachers during or after their major courses, participant J, a senior student, revealed that she needed to communicate with some teachers who helped her with the master’s program application, and that she also needed to communicate with her dissertation tutor. Participant I (also a senior) mentioned this motivation as well. She observed a year-by-year increase in her WTC in English because of her improvement in English vocabulary and proficiency.

Theme 3: International English Language Testing System (IELTS) or Graduate Management Admissions Test (GMAT)

Three students mentioned the need to take the IELTS or GMAT tests, which involve oral examinations. They regarded these tests as a positive factor in their WTC in English. Participant J made the following observation:

I think I spoke English most when I prepared for the IELTS exam in the summer vacation after the second semester of my junior year. I would talk to foreigners in the mock oral practice, and basically I would have a 15-min communication with foreigners every other day.

In her opinion, performing well on the IELTS would be useful for her master’s program application and future job hunting.

Theme 4: Need to improve interpersonal relationships

This theme described interviewees who noted extroverted behavior and an interest in engaging others in conversation. Participant D said she is an outgoing person, implying that it may not be challenging for her to talk to someone even in English. Her boyfriend, who had lived in Britain for a long time and spoke English regularly, may have provided additional motivation because she conversed with him in English. Participant F reported that she once volunteered to talk to one of her teachers because she liked that teacher. Participant H noted that she wouldn’t refuse to talk to a foreigner as long as he or she wanted to talk to her: “You can introduce to them China’s places with delicious food, or learn about their opinions on such issues as China’s weather and the reasons that they came here.”

Theme 5: Willingness to improve spoken English

Students whose words supported this theme wanted to improve their spoken English, not just for testing purposes, but due to an interest in improving their English speaking skills. Participant C viewed it as a breakthrough to take the initiative to start a conversation in English with a teacher who was able to speak Chinese. Participant H would take one-on-one English tutorials once a week, having a 30-min talk with a foreigner every time. Similarly, participant J thought her English proficiency could improve a lot if she could communicate with foreigners.

Theme 6: Current or potential job requirements

More than half of the interviewees realized the importance of using English in certain jobs. Participant F said English proficiency could set her apart, to some extent, from competitors when applying for an internship over the holidays, but she didn’t mention which aspect of English proficiency was required.

In contrast, participant J mentioned two specific situations that would require the use of English.

At work, first of all, English is a useful medium of communication when sometimes there comes a foreign customer. And I have friends who worked for an ad company and needed to translate some Chinese advertisement texts into authentic English, which is difficult without systematic study. And there is also the need to translate the Chinese name of a brand. For example, why “xicha” is translated into “Hey Tea?” I feel that a strong command of English is required for such kind of translation.

These two participants both focused on English proficiency; however, as far as participant K was concerned, a strong command of English was one thing, but the ability to use English as a tool to acquire information and knowledge also mattered a lot. When asked “How do you think about the role of English in your present or future life?” he replied that “people with GP [are] in great demand in the whole job market.”

#### Concerns Constraining Willingness to Communicate in Second Language

Theme 7: Lack of confidence in accent, grammar, vocabulary etc.

Participant C said it was inconvenient for her to express everything in English, since her lexical resources did not cover her communication needs in daily life. Participant D worried about whether her sentences were grammatically correct or authentic enough. For participant F, the biggest concern constraining her L2 WTC was insufficient fluency and accuracy. For participant G, when she was unable to comprehend what the teacher was talking about during class, she would not talk.

Theme 8: Peer pressure in out-of-class social contexts (e.g., being seen as showing-off and feeling awkward speaking English among Chinese-speakers)

Students’ lack of confidence could worsen when their peers expressed judgment of their accents or grammatical errors. Participant H described one such incident in the following excerpt:

Students around me seldom speak English, hardly ever. I think it is somewhat strange, because I think a lot of Chinese students, they pay special attention to English pronunciation and grammar. If you don’t pronounce it correctly, or if you have grammar mistakes, they would feel like laughing at you or something.

Additionally, since most students at the EMI university are native Chinese speakers who have been used to speaking Chinese for about 20 years, there seemed to be a default consensus to not speak English in daily life. Participant E said he used Chinese most of the time after class and concluded that “maybe it is the Chinese context that inhibits us from communicating in English, because it can be quite awkward.”

Participant K mentioned similar concerns when talking about the negative factors constraining L2 WTC: “It depends on the context and atmosphere. If everybody around you talks in Chinese, then there is no need to speak English. The use of English in such situation would be seen as showing off, right?” However, he added that he has been trying to get used to the English-speaking environment and improve his speaking skills, which increased his WTC in English. “When I get over the embarrassment, I become mentally prepared to be more willing to speak English than before.” After 4 years of experiencing an English environment, he said he generally holds a positive attitude toward communication in English.

Theme 9: Awareness of avoiding taboos

Two interviewees expressed worries about violating taboos. For instance, participant F explained, “sometimes I fear my questions are touchy or might disturb the private life of the people I want to ask. To avoid that, I would surf the Internet and find the answer by myself.” Likewise, participant H noted the fear that she “might violate their taboos unintentionally, which would make me a rude person in their eyes” constrained her WTC in English with foreigners.

Theme 10: Awareness of the western-centrism and promotion of their values

The concern noted in this theme might be an outcome of the course that the two interviewees once took. Both participant F and participant I, a sophomore and a senior, mentioned the US individualistic heroism.

When being asked about their understanding of GP and to give examples about GP, both participant F and participant I, a sophomore and a senior, mentioned the US individualistic heroism, an American value. Participant F thought individual heroism is kind of cultural export that helps the US economy. Participant I considered the US individualistic heroism as a part of American culture, which demonstrated his GP. They were able to understand this value from the perspective of a different culture. However, they did not have a clear idea whether their L2 WTC is constrained by it or not.

When the interviewer mentioned that there are few people around her speaking English, even though she was an English major, participant E said: “The low frequency of Chinese students speaking English partly represents a low popularity of the western-centrism, which is a good sign for China.” For him, L2 WTC is not necessary or very important. In his opinion, encouraging Asian people to speak English might be a means of promoting western centrism. When people are not obsessed with learning a foreign language, it shows that their own country is getting stronger. That may be what he meant “the good sign.”

#### Intercultural Cognition Facilitating Willingness to Communicate in Second Language

Theme 11: Possible needs in present life or when living abroad

Participant F thought of English as a necessary skill for her possible future daily life abroad, such as the inevitable conversations she would encounter while shopping or asking for directions.

Participant J, who planned to study in an English-speaking country for her master’s degree, also anticipated English-speaking scenarios such as talking to her roommates, who might be foreigners, or communicating with people in public places like shopping malls, airports, or bus stations. When it came to the role of English in her life, she reasoned that being able to use English could improve life quality: “Nowadays more and more products are entering [the] Chinese market, the instructions of which are all in English. If you have a good command of English, you can read and understand the ingredient list and know what is added to it.” Participant J added that she had been actively practicing using English.

Theme 12: Interests in the culture of English-speaking countries, such as movies, music, slangs, brain teasers

Two participants expressed a neutral attitude to the role curiosity about foreign cultures might play in their WTC in English. Participant G viewed these interests as sufficient but unnecessary to WTC in English. She enjoyed the feeling of speaking English fluently, which could be further improved if she was curious about foreign cultures. However, she didn’t think that watching American soap operas or listening to English songs would enhance her WTC in English. In other words, she thought her speaking may benefit from her WTC in English but not her listening. Participant H expressed her interest in the slang used in an American TV series, but she did not view a shared cultural background or overall knowledge of a culture as a motivation for her to communicate with people in English.

Participant E was indifferent to the effect that curiosity had on his English learning, which in his words was a burden. But his other opinions quoted in the following themes indicated that he seemed to have GP to some extent. He might represent a group of people who have GP but are not very willing to communicate in English. In contrast, participant J represents another group of people, who attach importance to having an interest in foreign cultures for the learning of any language:

I think the curiosity and attention to a foreign culture will immensely promote my willingness to learn about that culture or a language. For instance, when I was a child, my parents would show me some Disney cartoons, such as Lion King or Snow White. I fell in love with English because I liked those cartoons. During my primary school and middle school, I have watched some movies and dramas, all in English, and those I like would enhance my willingness to learn English deeply for the purpose of understanding the brain teasers.

Theme 13: Willingness to help foreigners

Participants E and J shared the experience of helping foreigners as a motivating factor for communicating in English. Participant E recounted telling a person from another country how to reach his destination in a Guangzhou subway station, and participant J once gave a foreigner instructions on how to obtain a COVID test at a hospital.

Theme 14: Awareness of the role of English as a lingua franca

Participant J and participant K, two seniors, both had the experience of using English as a medium to communicate with peers from other countries and saw it as a significant reason to use English. Participant J recounted the following situation: “There was a Korean in my group when I was a freshman, but he could not speak Chinese, so I always communicated with him in English.” She explained that, at that time, she realized the importance of learning English, noting, “I always hold a positive attitude toward English learning.”

Similarly, participant K noted the usefulness of English as a common language among people from different countries:

English is the first choice in some conditions where many non-native speakers get together. For example, Korean or Japanese would use English to start a conversation in the first meetings, and when we realize they cannot speak Chinese, we will use English, too. After all, English is a widely used language and is taken as a second language in most countries.

Theme 15: Accessing firsthand sources of information, such as news or comments on foreign websites

Participants E and G mentioned the errors of translated news originally published on foreign websites, and showed their willingness to read the original English news or find the firsthand materials. For participant I, he believed that he could only understand foreign cultures in depth by communicating with the local people of those countries firsthand, rather than being told about them in China.

Theme 16: Understanding GP (global perspective)

Interviewees from different grades in the EMI university seemed to have a similar understanding of global perspective.

Participant A (a freshman) stated, “I think global perspective means looking at the whole world with a broader view and in the longer run, instead of just the current situation in China.” Participant E (a sophomore) used similar terms:

A global perspective is one that considers other countries when you think about something. For example, going to other countries is also a choice for attending college, and we needn’t limit our choices to the universities in mainland China.

Participant G (a junior), expressed the opinion that a “global perspective stands for an advanced perspective, with which we can judge things from the point of view of other countries’ people, especially some international issues.” Participant I (a senior): “I think global perspective refers to the care about other countries’ culture or events, such as the formation of American mainstream ideology, and individualistic heroism.”

The excerpts above demonstrate that GP is not limited to students from a particular grade. In fact, it may be equally important for students to have knowledge about and understanding of international affairs and foreign cultures throughout the learning process. From an early age, learners can embrace the world and its various cultures and, thus, form a global perspective, which in turn, may facilitate their WTC in English.

## Discussion

The findings of the interview data echo the results of the questionnaire. For research question one, the answers of interviewees from years 1 to 4 have much in common, which indicates that participants of different groups share similar GP and L2 WTC. Though the scores of the seniors’ GP and L2 WTC were the highest, chances are that seniors used English less than in previous years because they had fewer courses to take.

For research question two, some students gave similar definitions for GP when asked about their understanding of it and reasoned that curiosity about foreign cultures could help them learn English, which suggests that a positive correlation might exist between GP and L2 WTC.

### Similarities Between the Findings of This Study and Previous Ones

#### Factors Driving or Constraining Willingness to Communicate in Second Language

Among the five positive factors contributing to the interviewees’ WTC in English, two reasons were frequently mentioned by interviewees from different grades. The first one was the course requirement. Nearly all participants put this reason at the top when asked about what makes them speak English. Freshmen were still trying to use English in class, while interviewees of other three grades had more or less gotten used to it. EMI courses seemed to be effective in cultivating students’ L2 WTC. The second reason students cited was for the IELTS or GMAT preparation; these tests are indispensable in the case of applying for master’s programs abroad. It was not only juniors and seniors who were preparing for or taking these examinations; several freshmen and sophomores also considered such needs, which indicates that students are more motivated to learn English if they are clear about English-learning goals. This finding is congruent with Fang et al. (2020) assertion on the relationship between self-actualization and WTC in English. It also lends support to previous researchers’ findings on the positive impacts of learners’ motivation on WTC in English (Lee and Lee, 2019; Shirvan et al., 2019).

Among the four negative factors constraining interviewees’ WTC in English, the first two are consistent with previous researchers’ findings that self-confidence directly influenced EFL students’ L2 WTC (Yashima, 2002; Peng, 2014; Peng and Xie, 2014; Shirvan et al., 2019). If English is merely regarded a knowledge-based skill, and grammar and accent are overemphasized in class, students may be reluctant to speak English due to a lack of confidence in their proficiency or the fear of being laughed at by their peers. Themes 8 and 9, awareness of avoiding taboos and awareness of the western-centrism, could prevent students from communicating with foreigners. At the same time, such concerns reflect the learners’ awareness of cultural differences, which in part belongs to the connotation of global perspective (Zhang and Zhu, 2020).

Students whose answers reflected the four factors in the third type, intercultural cognition facilitating L2 WTC, could be regarded as having realized the fun and importance of communicating—not limiting English to a subject or an exam, but treating it as a tool to communicate or to look at the world (Fang et al., 2020; Zhang et al., 2020).

By and large, some findings were consistent with the concept of individual factors (Peng, 2019; Alimorad and Farahmand, 2021; Ma et al., 2022) or within-person variability (Zhang et al., 2019), such as themes 3, 6, 11, and 13, and most of the rest are in line with the contextual/environmental factors (Peng, 2019; Alimorad and Farahmand, 2021; Ma et al., 2022) and between-person variability (Zhang et al., 2019; Zhang et al., 2020). While the previous studies mainly focused on in-class factors that influenced students’ L2 WTC, the current study discovered some additional out-of-class factors, like themes 5, 12, and 13, which show students’ initiative to use English in real-life situations, and to some extent confirm previous researchers’ finding that WTC is socio-culturally constructed as a function of the interaction of individual and environmental factors both inside and beyond the classroom walls (Peng, 2019; Alimorad and Farahmand, 2021; Ma et al., 2022).

### Differences Between the Findings of This Study and Previous Ones

Few previous studies have focused solely on the relationship between GP and L2 WTC, except for Fang et al. (2020) study, so the differences between their findings and those of the current study will be discussed below.

Firstly, the number of questionnaire and interview participants has increased substantially in this study. In Fang et al. (2020) study, there were 114 questionnaire participants and 7 interviewees; whereas in this study the numbers are 315 and 11, respectively. The enlarged data set helped to uncover more information to explore the correlation between GP and L2 WTC. For example, in the study, due to the enlarged interview data, we could identify positive and negative factors. While there are five conceptual themes (global cognition, self-actualization, intercultural experience, WTC in English, and English learning) in Fang et al. (2020) study, the current study identified 16 detailed themes, each presenting a specific issue. When analyzing the interview data, the excerpts in Fang et al. (2020) study were analyzed under three subtitles: global cognition and WTC in English, self-actualization and WTC in English, intercultural experience and WTC in English, in which the relationship between the factors and L2 WTC was not clear; in comparison, the excerpts in this study were analyzed under the three subtitles showing the positive or negative influence.

As for the research context, this study was conducted at an EMI university where nearly all classes were conducted in English from year 1 regardless of students’ English proficiency, while Fang et al. (2020) study was conducted at an English Language Center (ELC) of a university located in southeast China where participants’ English proficiency level was the basis for grouping the students into different levels of English classes. Fang et al. (2020) called for further research on students taught by foreign or international teachers, and the current study addressed this gap by examining students of an EMI university where there were many international teachers.

As for the participants’ global cognition level, in contrast to the interviewees in Fang et al. (2020) study who did not have strong global cognition, many interviewees in this study seem to have different levels of GP, which might be the outcome of immersion EMI courses. Furthermore, it was confirmed that a global cognition may enhance one’s L2 WTC in the cases of participants H, J, K who had experiences of taking initiative for intercultural communication with foreigners, though not necessary in the cases of participant E who cared about international issues but didn’t have much desire to communicate in English. Additionally, in the case of participant K, an argument might be made that L2 WTC enhanced his GP; this participant explained that after getting over the concerns constraining L2 WTC, such as out-of-class context and a lack of confidence, he became more willing to communicate in English to meet the expectations of the job market, which increasingly requires job seekers to have high level of global perspective.

### Relating the Findings Back to Theories

We would like to further discuss our findings in relation to the pertaining theories. First of all, we would examine our interviewees’ GP in light of the theory of Braskamp et al. (2014), who asserted that the cognitive development of GP includes the awareness of and the ability to seek foundational truths by viewing complicated and comprehensive knowledge and considering multiple cultural perspectives. Participants E, G, and I’s answers reflect their intention and attempts to seek comprehensive facts, because they mentioned their needs in accessing the first-hand sources of information in order to get closer to the truth. Their L2 WTC varied, however, because of the different role of English in their opinion. Participants E and I may view English simply as a required subject, while G described having had an interest in spoken English from a young age.

The developmental end of a journey along the intrapersonal domain involves “a sense of self-direction and purpose in one’s life, becoming more self-aware of one’s strengths, values, personal characteristics and sense of self, as well as viewing one’s development in terms of a clearer self-identity” (Braskamp et al., 2014). These qualities were reflected in the interviewee excerpts of participants B, C, F, H, J, and K. Participant B realized that his introverted personality hindered his WTC either in Chinese or English. C described becoming aware of her insufficient oral expression and had begun to make efforts to improve. Meanwhile, F and J alluded to preparing for the IELTS for future further study and job hunting.

Braskamp et al. (2014) define interpersonal development as centering on an individual’s “willingness to interact with persons with different social norms and cultural backgrounds, acceptance of others, and being comfortable when relating to others.” Interview excerpts from participants J and K supported this view. Both of them reported interacting with group members from foreign countries, and expressed a high level of WTC in English. They had realized the importance of English as a medium of communication in real world use, which confirms that intercultural experience may also enhance WTC (Fang and Baker, 2017).

Furthermore, the interview findings seem to lend support to Yashima’s (2002) theory of international posture. Some themes of the three factors influencing L2 WTC are congruent to the four aspects of international posture: namely, interest in international vocation/activities (IVO), international friendship orientation (IFO), interest IFA and intergroup AAT.

As far as IVO is concerned, theme 11 (learners’ possible needs when living abroad) and theme 14 (awareness of the role of the English as a lingua franca) are confirming it. Participant F, J, K expressed their needs to use English in international activities.

IFO is supported by theme 4 (need to improve interpersonal relationships) and theme13 (willingness to help foreigners). Participant D was willing to talk in English with her English-speaking boyfriend; participant F used speaking English as a way to make friends with her adored foreign teacher; participant E and J once helped foreigners in daily life, where English was a medium of communication.

IFA is validated by theme 10 (awareness of the western-centrism and promotion of their values), theme 12 (interests in the culture of English-speaking countries) and theme 15 (accessing firsthand sources of information). From talking about western values such as western ethnocentrism and individualistic heroism, to being interested in western cultural products such as movies and songs, from volunteering themselves to searching and reading international news reports, to seeking for authentic foreign cultures, participants F, I, E, G showed their knowledge and awareness of foreign events and cultures.

In terms of AAT, theme 9 (awareness of avoiding taboos) aligned with it. Since participants F and H had realized the cultural differences, they chose to respect foreigners with possible different cultural norms that they did not know and be careful not to make the foreigners feel uncomfortable.

To sum up, the factors influencing L2 WTC are also supporting global perspective (GP), which to some extent demonstrates that L2 WTC and GP are correlated.

### Pedagogical Implications

From the above discussion, several pedagogical implications can be drawn in the context of EMI universities:

(1) Create a non-threatening English-speaking environment and encourage students to communicate in English in and outside the classroom (Xiao and Zhao, 2020, 2022). Several interviewees recognized that they would be more willing to speak English if people around them were doing the same thing. It is optimal for students to get used to speaking English as if they were speaking their native language.

(2) Emphasize the L2 WTC and content over pronunciation and fluency. Many students expressed their concerns in this regard. For instance, participant B was extremely reluctant to communicate in English because he thought his English was poor and felt uncomfortable being forced to do so. Instructors need to make students feel comfortable about making mistakes when using English to communicate. Students do not need to have a perfect command of English before they open their mouths to speak; teachers can emphasize that perfection is hard to achieve and only practice makes perfect. It’s more important for the learners to be able to communicate in English rather than speaking English with perfect grammar and a perfect accent.

(3) Educate students about GP and L2 WTC. The teachers may expose the students to current events and news stories, such as American presidential election, Huawei Chip sanctions, COVID herd immunity etc. Furthermore, the teachers may explain GP and L2 WTC in relation to the news before engaging the students in the discussion, analysis, and writing tasks about those news, in an attempt to foster students’ awareness and development of GP and L2 WTC. A multilingual environment should be multicultural with open-minded students who embrace the linguistic diversity and the diversity of ideas and perspectives (Zhao and Xiao, 2020, 2021).

Additionally, universities could provide specialized guidance or facilities for students with specific needs, such as practicing English speaking, seeking advice on graduate school application abroad, and job searches.

## Conclusion

Using two questionnaires and interviews, this study examined the correlation between GP and WTC in English at an EMI university in mainland China. It found that there were no significant differences in the mean scores of GP or L2 WTC among students from years 1 to 4, but a moderate positive correlation between GP and L2 WTC did exist according to the questionnaire data analysis.

Some limitations of the current study need to be acknowledged. First, the sample sizes of the questionnaires (*n* = 315) and interviews (*n* = 11) were relatively small. A much larger sample size may yield more valid and reliable findings. In addition, the study only employed questionnaires and interviews to collect data. Different sources of data can be added to future research, such as class observations, reflective journals, and test scores, etc.

For future research, we offer the following suggestions. First, the theoretical foundation of the relationship between GP and WTC needs to be improved. Braskamp et al. (2014) defined GP from a macroscopical view, and more details related to daily life need to be added in order to make the standard less abstract and more specific, so that it is easier for other researchers to understand. Second, the correlation between GP and WTC could be further examined with different student populations, such as English majors, different non-English majors, and graduate students, at different types of universities across different countries. Third, comparative studies can be conducted between an EMI university and a non-EMI traditional university in China. Since students in this study were mainly taught by international and Hong Kong professors, future studies could compare students taught by mainland Chinese professors and those instructed by international and Hong Kong professors. Finally, the questionnaire items and interview questions in this study can be revised to suit various specific research contexts better.

## Data Availability Statement

The raw data supporting the conclusions of this article will be made available by the authors, without undue reservation.

## Ethics Statement

The studies involving human participants were reviewed and approved by the Hunan Normal University. The patients/participants provided their written informed consent to participate in this study.

## Author Contributions

YX was responsible for the conceptualization, methods, survey data collection and data analysis, revisions, and editing of the manuscript. XQ wrote the first draft and was involved in interview data collection and data analysis, and revising the manuscript. Both authors contributed to the article and approved the submitted version.

## Conflict of Interest

The authors declare that the research was conducted in the absence of any commercial or financial relationships that could be construed as a potential conflict of interest.

## Publisher’s Note

All claims expressed in this article are solely those of the authors and do not necessarily represent those of their affiliated organizations, or those of the publisher, the editors and the reviewers. Any product that may be evaluated in this article, or claim that may be made by its manufacturer, is not guaranteed or endorsed by the publisher.
